# Current Routine Testosterone Immunoassays Are Unsuitable for Lowering the General Castration Cutoff Recommendation to <0.7 nmol/l (20 ng/dl)

**DOI:** 10.1016/j.euros.2022.12.009

**Published:** 2023-01-09

**Authors:** Huub H. van Rossum, Lennart J. van Winden, Andries M. Bergman, Henk G. van der Poel

**Affiliations:** a Department of Laboratory Medicine, Netherlands Cancer Institute, Amsterdam, The Netherlands; b Department of Medical Oncology, The Netherlands Cancer Institute, Amsterdam, The Netherlands; c Department of Urology, The Netherlands Cancer Institute, Amsterdam, The Netherlands; d Department of Urology, Amsterdam University Medical Center, Amsterdam, The Netherlands

## Abstract

Testosterone measurements are essential in the management of patients with prostate cancer undergoing castration and androgen deprivation therapy. There has been an ongoing discussion on the testosterone castration cutoff (TCC), with the primary focus on large cohort studies in which the testosterone measurement system was not specified or studies that used individual testosterone measurement systems. Here we present a post hoc analysis of a study comparing testosterone measurement systems in a cohort of 120 castrated patients with prostate cancer. We investigated the suitability of general, measurement system–independent, TCC values recommended in all clinical guidelines. We show that the four testosterone immunoassays commonly used are unsuitable to support lowering of TCC to 0.7 nmol/l (20 ng/dl) testosterone, since testosterone levels are falsely quantified as higher than this cutoff in 4.2–29.2% of the castrated cohort, depending on the testosterone immunoassay used. When using 1.0 nmol/l (30 ng/dl) as the TCC for the Beckman immunoassay, 13.3% of the results were falsely quantified as being higher than this value. The results suggest that the measurement systems used in current practice do not support lowering the TCC to 0.7 nmol/l. Furthermore, a more local, immunoassay-dependent TCC should be considered.

**Patient summary:**

Patients with advanced prostate cancer who are treated to reduce their testosterone to a castration level are monitored using testosterone measurements. The testing systems currently used for measurement do not support lowering of the testosterone cutoff value to 0.7 nmol/l. Testosterone cutoff values to define castration status should preferably be based on the measurement system in local use.

Even 80 yr after the discovery of its effects in prostate cancer (PC), castration remains the major pillar of treatment for advanced PC and a concomitant or adjuvant treatment following external beam radiotherapy to the prostate. The efficacy of castration treatment, which generally involves administration of luteinizing hormone–releasing hormone agonists or antagonists, is determined via measurement of circulating testosterone concentrations. Castration-resistant PC is defined as progressive disease while having sufficiently low serum testosterone concentrations that reflect adequate castration. The generally recommended testosterone castration cutoff (TCC) of 1.7 nmol/l (50 ng/dl) to define adequate castration treatment was initially based on the lowest possible detection level of historical testosterone measurement systems and has largely remained unchanged in clinical guidelines [1], [2], [3]. Recent investigations have shown that lower testosterone castration levels correlate with better survival. In particular, it was found that a TCC of 0.7 nmol/l (20 ng/dl) was associated with survival prognosis and this was therefore suggested as a new TCC for adequate testosterone deprivation [4]. Observations using single testosterone measurement systems for surgically castrated men indicated lower TCCs, which prompted the European Association of Urology to recommend a TCC of 1.0 nmol/l (30 ng/dl) [5], [6].

Our group recently reported a method comparison of the four most widely used immunoassay-based testosterone measurement systems and one liquid chromatography-tandem mass spectrometry (LC-MS/MS) measurement system in a study using sera from 120 castrated patients with PC [7]. LC-MS/MS technology is considered the reference methodology for steroid and testosterone analysis owing to its superior analytical sensitivity and specificity and accuracy, especially for the lower analytical range (<2.0 nmol/l) [1], [8]. Correlations between measurement systems were determined. The correlation coefficients observed ranged from 0.15 to 0.58 for correlations between the immunoassays and the LC-MS/MS measurement system, and from 0.23 to 0.76 for between-immunoassay correlations. This is very poor from an analytical perspective, since it is claimed that these assays measure the same, well-defined measurand: testosterone. Using the results obtained, we determined the measurement system–specific upper limit (97.5th percentile) for the castrate population, which ranged from 0.495 nmol/l for the LC-MS/MS method to 1.32 nmol/l for one of the immunoassays [7].

As a follow-up, we assessed the suitability of general, measurement system–independent TCC values of 0.7 nmol/l, 1.0 nmol/l, and 1.7 nmol/l for the different testosterone measurement systems. To this end, we calculated the percentage of testosterone results within the castration criteria for each measurement system (Table 1). For a TCC of ≤1.7 nmol/l, all testosterone results from all measurement systems met this criterion. However, for a TCC of <0.7 nmol/l, the percentage of testosterone results meeting this criterion differed among the measurement systems. In laboratories using the Abbott, Beckman, Roche, and Siemens immunoassay analyzers, the castration status of 13.7%, 29.2%, 4.2%, and 8.3% of the patient cohort, respectively, would be categorized as inadequate. For the LC-MS/MS measurement system, all testosterone measurements were below this cutoff. It should be noted that these different results were observed for measurements on exactly the same serum samples. This large method dependence for the insufficient castration rate when using a TCC of 0.7 nmol/l, together with the lack of correlation and accuracy previously observed [7], significantly compromises the use of such a low general TCC since the measurement system used significantly influences the measurement outcome and corresponding clinical diagnosis.

**Table 1:** Testosterone results for different castration cutoffs and immunoassay-specific cutoffs

Castration cutoff	Testosterone results meeting the cutoff criterion (%)				
	LC-MS/MS	Abbott	Beckman	Roche	Siemens
<0.7 nmol/l (20 ng/dl)	100	86.3	70.8	95.8	91.7
<1.0 nmol/l (30 ng/dl) a	100	98.3	86.7	99.2	100
≤1.7 nmol/l (50 ng/dl)	100	100	100	100	100
**Upper percentiles**^b^					
95th percentile	0.47	0.91	1.25	0.66	0.70
(90% CI)	(0.47–0.60)	(0.82–1.10)	(1.24–1.35)	(0.61–1.06)	(0.70–0.90)
97.5th percentile	0.50	1.02	1.32	0.91	0.80
(90% CI)	(0.47–0.60)	(0.61–1.15)	(1.24–1.35)	(0.61–1.06)	(0.70–0.90)
99th percentile	0.58	1.40	1.35	1.03	0.90
(90% CI)	(0.47–0.60)	(1.38–1.81)	(1.24–1.35)	(0.61–1.06)	(0.70–0.90)

CI = confidence interval; LC-MS/MS = liquid chromatography-tandem mass spectrometry.

a Conversion between units would result in 1.0 nmol/l → 29 ng/dl, but 30 ng/dl → 1.0 nmol/l because of rounding, so 30 ng/dl was used for reasons of convenience.

b The 95th, 97.5th, and 99th percentiles and the corresponding 90% CIs as previously determined for a castrated prostate cancer population are presented. Testosterone cutoff concentrations in nmol/l were adapted from van Winden et al [7].

Our results demonstrate that from an analytical perspective, current routine testosterone immunoassays are unable to quantify castrate testosterone levels in castrated men and hamper implementation of a lower TCC of 0.7 nmol/L in the clinic. Moreover, a TCC of 1.0 nmol/l might be inappropriate for laboratories that use the Beckman immunoassay. Measurement system–specific cutoff values might be an alternative approach to lower and optimize TCC. This is probably already being applied to some degree in local settings when the lower limit of quantitation of the measurement system or an adjusted lower cutoff based on practical experience is used.

  ***Conflicts of interest***: Huub H. van Rossum owns stock in Huvaros B.V. and SelfSafeSure Blood Collections B.V., and has received grants, contracts, or consulting fees from Abbott, Roche, Siemens, and Sysmex. Andries M. Bergman has received grants, contracts, or consulting fees from Bayer, Sanofi, Astellas, and Janssen. Lennart J. van Winden and Henk G. van der Poel have nothing to disclose.
